# Changes in hip and knee arthroplasty practice post-COVID-19 in the English NHS: a retrospective analysis of hospital episode statistics data

**DOI:** 10.1308/rcsann.2024.0100

**Published:** 2024-11-21

**Authors:** TW Wainwright, T Immins, RG Middleton

**Affiliations:** ^1^ Bournemouth University, UK; ^2^ University Hospitals Dorset NHS Foundation Trust, UK

**Keywords:** Hip arthroplasty, Knee arthroplasty, COVID-19

## Abstract

**Background:**

The COVID-19 pandemic significantly reduced hip and knee arthroplasty surgeries across the English NHS. With the resumption of regular operations postpandemic, efforts have been made to address the surgical backlog by maximising capacity. This study assesses the current activity rates of hip and knee arthroplasty in the NHS and their return to prepandemic levels.

**Methods:**

We analysed hospital episode statistics from all English NHS providers of hip and knee arthroplasty from 1 April 2018 to 31 March 2023. Variables such as activity, location of surgery, length of stay and readmission rates were examined.

**Results:**

Data for 706,772 hip and knee arthroplasty surgeries show that overall activity from 1 April 2022 to 31 March 2023 has decreased by 8.8% compared with the initial year of the study. During the last year, 38.4% of surgeries were performed in the independent sector, an increase from 29.6% in the first year.

**Discussion:**

The postpandemic recovery phase has seen a strategic shift of surgeries to the independent sector, which helps reduce backlogs but poses risks to the role of the NHS in surgical training and innovation. This redistribution has immediate benefits for patient care but may impede trainee development and weaken research capabilities due to the lack of infrastructure in independent sectors. To maintain its leading role in orthopaedic care, the NHS needs to explore innovative solutions and strategic partnerships, incorporating advanced technologies and new training methods to adapt to the evolving healthcare landscape.

## Background

The healthcare system in the UK is predominantly publicly funded and delivered through the NHS, which provides free healthcare to residents at the point of use. The NHS is funded primarily through taxation and is responsible for most healthcare provision. Alongside the NHS, the independent sector has historically provided healthcare to self-pay and privately insured patients for elective surgical procedures. Primary hip arthroplasty (THA), and primary knee arthroplasty (TKA) are high-volume elective surgical procedures in the English NHS^1^ and, before the COVID-19 pandemic, the aging population was contributing to a year-on-year increase in the number of procedures performed.^1^ However, the COVID-19 pandemic has had an obvious and significant impact on healthcare systems across the world, including the NHS, with a pause and reduction to elective surgery activity, and then a phased return to elective operating for hospitals. This has meant that one of the major sequelae following the COVID-19 pandemic is an elective surgery backlog and a subsequent large volume of THA and TKA patients to treat.^2^

The post-COVID-19 pandemic period therefore poses a capacity challenge to the NHS, with the government proposing that the NHS in England partners with independent sector providers to overcome this.^3^ This proposal comes following the ability and willingness of the independent sector to work collaboratively with the NHS towards a common goal during the COVID-19 pandemic.^3^ To that end, the independent sector now currently delivers 6% of all diagnostic tests, and 9% of all appointments or treatments that completed a patient pathway and removed patients from a waiting list in 2022–2023.^4^ However, data have not yet been presented to analyse the degree to which elective THA and TKA operating has returned to the NHS, and to what extent this is being completed in NHS hospitals or the independent sector. The outcome of this analysis was therefore to examine current trends in activity rate and location of THA and TKA in the NHS and examine to what degree it has returned to prepandemic levels.

## Methods

This is a retrospective analysis of THA and TKA procedures performed by the English NHS and is reported using the reporting of studies conducted using observational routinely collected health data (RECORD) checklist.^5^ The analysis was performed using hospital episode statistics (HES) data collected between 1 April 2018 and 31 March 2023. HES data are a valuable resource for research and allow for comparisons of outcomes.^6^ The HES data were accessed via the Telstra Health UK Limited (Dr Foster) Health Intelligence Portal,^7^ an online tool that receives clean HES data from the Health and Social Care Information Centre that can be used for presenting and comparing healthcare data. In accordance with NHS Health Research Authority guidance, ethical approval was not required for this study.^8^

The analysis tool in the Health Intelligence Portal was used to extract the data. All NHS and independent providers of NHS procedures were included in the analysis. All elective primary THA and TKA procedures were included. The primary outcome measure (the number of spells) and the location of surgery (NHS hospital or independent provider) were calculated by including all patients between 1 April 2018 and 31 March 2023. The number of spells extracted from the HES data represents the number of procedures performed.

## Results

Table 1 summarises the number of spells in the NHS in the years March 2018/April 2019 to March 2022/April 2023, divided into NHS and independent settings. There was a total of 706,772 spells, covering primary THA and TKA, within this time period.

**Table 1:** NHS THA and TKA procedures by location April 2018 to March 2023

Year	April 2018/March 2019		April 2019/March 2020		April 2020/March 2021		April 2021/March 2022		April 2022/March 2023		Total
	Spells	%	Spells	%	Spells	%	Spells	%	Spells	%	Spells
Independent	51,580	29.6	52,535	31.0	19,985	32.8	51,130	35.8	61,110	38.4	236,340
NHS	122,942	70.4	116,933	69.0	40,861	67.2	91,659	64.2	98,037	61.6	470,432
Total	174,522		169,468		60,846		142,789		159,147		706,772

THA = primary hip arthroplasty; TKA = primary knee arthroplasty

Across all NHS providers, total activity level for THA and TKA has not yet returned to pre-COVID-19 levels. In the first year of analysis, 1 April 2018–31 March 2019, 174,522 procedures were performed, compared with 159,147 in the final year of analysis (1 April 2022–31 March 2023). This represents a decrease of 8.8% in activity, although annual surgical volume is increasing following the most COVID-19-affected year, 2020–2021, when only 60,846 procedures were performed.

**Figure 1:**
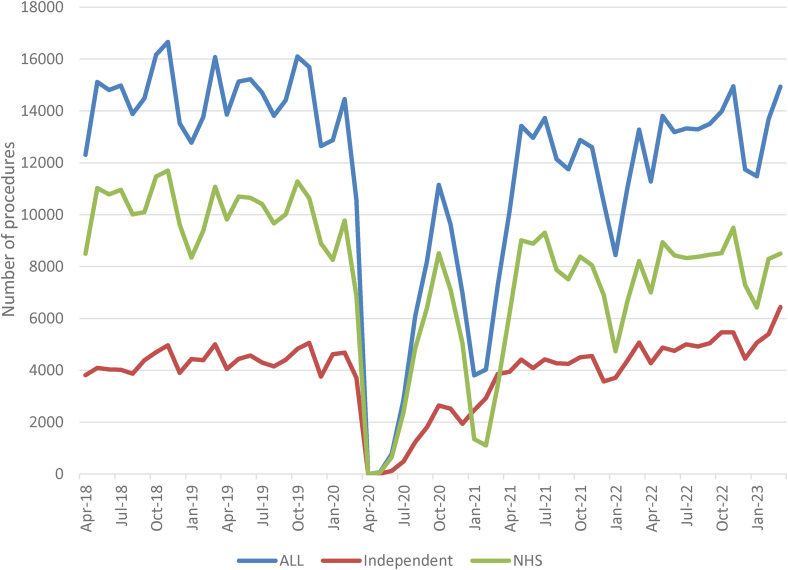
Total NHS THA and TKA procedures, April 2018–March 2023, broken down by location. THA = primary hip arthroplasty; TKA = primary knee arthroplasty

**Figure 2:**
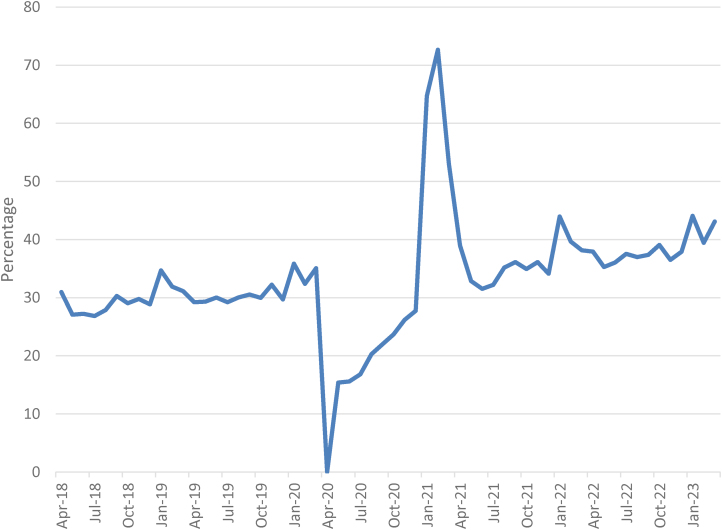
Percentage of NHS THA and TKA procedures performed in independent locations, April 2018–March 2023. THA = primary hip arthroplasty; TKA = primary knee arthroplasty

Figure 1 is a graph with the total number of procedures by month for the four years, and the breakdown by location – NHS or independent. It shows that, when comparing 2018–2019 with 2022–2023, there has been a shift in the location of surgery, with an increasing number of procedures now being performed in an independent location. Figure 2 shows the percentage of procedures performed in an independent location by month over the four years. In April 2018, 31.0% of spells were performed at an independent location, and in March 2023 this had increased to 43.1%.

## Discussion

The ongoing recovery of THA and TKA volumes in the NHS to prepandemic levels shows the profound and lasting impact of the COVID-19 pandemic on elective surgeries. Despite an ongoing increase in annual NHS surgical volumes following the pandemic, this recovery is driven primarily by activity in the independent sector, underscoring a significant post-COVID-19 pivot in the location of orthopaedic care delivery. This is undoubtedly benefiting current patients by not only providing the additional capacity to operate on the large numbers of patients on the waiting list, but also to provide these operations in high-quality facilities with consultant-level care. However, this shift is not without potential future consequence, particularly as the capacity of NHS hospitals to perform these critical procedures continues to decline. This decrease in NHS surgical capacity has multifaceted repercussions, extending beyond the reduced activity, as it has potential future impacts on the foundational pillars of surgical education, training and research.

### Impact on surgical education and training

The migration of routine orthopaedic operations to independent hospitals presents a paradox in surgical education in the NHS. Traditionally, the NHS has provided a comprehensive pathway for orthopaedic education, from undergraduate training to postgraduate specialisation. However, the current practice shift, as presented in this paper, jeopardises this continuum, potentially limiting trainees’ exposure to the required volume and range of surgical experiences. This limitation is particularly concerning for routine procedures, which are crucial for honing basic surgical skills, and are also the ones most often outsourced to independent sector hospitals. Of the operations that remain in NHS hospitals, the proportion of joints that are complex will therefore increase, requiring more consultant operating and cases that are harder to use as teaching cases. The reduction in training opportunities in NHS settings could therefore potentially dilute the quality of training and prolong the timeline for trainee progression, unless innovative solutions, such as integrating training in the independent sector, are explored and implemented effectively.

### Research and innovation dilemma

Moreover, the shift towards the independent sector raises significant concerns regarding research and innovation in orthopaedic care. NHS hospitals have traditionally been hubs for clinical research, contributing significantly to advancements in patient care. Reducing elective surgeries in NHS facilities could stifle these research endeavours, given the very limited research infrastructure in independent hospitals. This scenario risks the future development of evidence-based practices and innovations in orthopaedic care, potentially impacting future patient outcomes.

### Strategic responses and future directions

To address these challenges, there is an imperative need for strategic partnerships and innovative training models. Collaborations between NHS trusts, academic institutions and independent providers could offer a multifaceted solution, ensuring the comprehensive training experiences needed for future surgeons. For example, new technology such as robotic surgery is being performed largely in the UK private sector, and is predicted to become a standard of care in the future. However, surgical trainees in the NHS may have no experience of this developing technology, and so training is not just about numbers of procedures, but also about exposing trainees to emerging technologies such as robotics, and so training for future surgeons could be improved by formally incorporating experience in the independent sector as part of recognised training. Moreover, leveraging modern technologies such as simulation and virtual reality could mitigate resource constraints and enhance educational outcomes.

In parallel, establishing formal research alliances across these entities could foster a conducive environment for orthopaedic research, ensuring that innovation and evidence-based practice continue to thrive. Presently, a major issue facing research teams is that patients are recruited for trials in the NHS but then must be withdrawn when their subsequent surgery is transferred to the independent sector. Therefore, if routine joint replacement continues to be increasingly done in the independent sector, research collaborations and shared ways of working are required to allow patients to continue to be part of research trials even if transfer from the NHS to the independent sector occurs.

## Conclusion

In conclusion, while the independent sector has played a crucial role in sustaining THA and TKA surgeries during the pandemic’s aftermath, evaluating and addressing the long-term implications of this shift is essential. Balancing service delivery with education, training and research priorities requires a concerted effort and strategic foresight, ensuring that the NHS can continue to provide world-class orthopaedic care in an evolving healthcare landscape.

## Declarations

### Availability of supporting data

The dataset supporting the conclusions of this article is available on request.
